# Can serum progesterone concentration direct a fresh or freeze-all transfer strategy in the first in vitro fertilisation cycle?

**DOI:** 10.1007/s10815-024-03103-y

**Published:** 2024-04-03

**Authors:** Sarah Hunt, Jing Liu, Pulin Luo, Ying Zhong, Ben W. Mol, Ling Chi, Rui Wang

**Affiliations:** 1 Department of Obstetrics and Gynaecology, School of Clinical Sciences at Monash Health, Monash University, Clayton, Victoria Australia; 2 Monash Womens, Monash Health, Clayton, Victoria Australia; 3 Monash IVF, Richmond, Victoria Australia; 4 Chengdu Xinan Gynecology Hospital, Chengdu, Sichuan China; 5 Aberdeen Centre for Women’s Health Research, Institute of Applied Health Sciences, School of Medicine, Medical Sciences and Nutrition, University of Aberdeen, Aberdeen, UK

**Keywords:** Freeze all, Embryo transfer, IVF, Progesterone, Live birth

## Abstract

**Purpose:**

To examine the interaction between serum progesterone concentration on the trigger day and choice of freeze-all and fresh transfer strategies on live birth in an unselected population as well as in patients over 35 years old.

**Methods:**

We performed a retrospective cohort study of 26,661 patients commencing their first IVF cycle in a large fertility centre between 2015 and 2019, including 4687 patients over 35 years old. We performed a multivariable fractional polynomial interaction analysis within a logistic regression model to investigate the interaction between serum progesterone concentration and the choice of freeze-all or fresh transfer strategy following the first transfer.

**Results:**

15,539 patients underwent a fresh embryo transfer and 11,122 underwent a freeze-all strategy in their first IVF cycle. The freeze-all group had a higher live birth rate compared to the fresh group (43.9% vs 40.3%). After adjusting for confounding factors, there was a positive interaction between serum progesterone concentrations and the choice of a freeze-all versus fresh embryo transfer on live birth (*p* for interaction 0.0001), with a larger magnitude of effect when progesterone concentration was higher. Such an interaction was also observed in patients over 35 years old (*p* for interaction 0.01), but the treatment effect curve over progesterone concentrations was almost flat.

**Conclusions:**

In an unselected population, frozen transfer is associated with greater chances of live birth, especially in patients with higher serum progesterone concentration. In patients over 35 years old, the benefit of a freeze-all policy appears small across all serum progesterone concentrations.

**Supplementary Information:**

The online version contains supplementary material available at 10.1007/s10815-024-03103-y.

## Introduction

Historically, most fertility clinics worldwide have practiced fresh embryo transfer, with frozen transfer of surplus embryos. Fresh embryo transfer is associated with increased chances ovarian hyperstimulation and studies suggest that there are differences in the resulting pregnancy [1–3]. These differences are thought to potentially reflect differences in placentation which result from the altered hormonal milieu and endometrial gene expression, characteristic of these treatment modalities [4, 5]. This, coupled with the advances in vitrification technology, has led to the introduction of freeze-all embryo transfer to clinical practice. Recognition of the potential impacts of transfer strategy on perinatal outcome as well as a move to a personalised approach to treatment has led to a shift in practice where frozen transfer is increasingly performed. Accordingly, the clinical effectiveness of one transfer strategy over the other has been the subject of many recent large randomised control studies [6–9].

A large multicentre trial found that for patients with polycystic ovary syndrome (PCOS), a freeze-all embryo transfer in the first cycle of treatment was associated with increased live birth rates [8]. The same group went on to report on the outcomes of patients who were normal ovulatory and found that freeze-all and fresh transfer were comparable with respect to live birth rate in the first transfer cycle [6]. This finding was confirmed in a second large study [7]. A subsequent multicentre trial, however, found that in ovulatory patients with good prognosis, frozen single blastocyst transfer was associated with a 10% increase in pregnancy rate (40% vs 50%) [9]. More recently published European studies have yielded similarly conflicting results. Stormlund et al. reported no difference in live birth rate for ovulatory patients when human chorionic gonadotrophin (HCG) trigger and fresh transfer was compared with agonist trigger and freeze all [10]. Wong et al. however reported a significant reduction in cumulative live birth at 12 months from freeze all as compared to fresh transfer. The resulting clinical equipoise has led to consideration of cycle specific factors which may effectively direct transfer strategy [11].

The role of progesterone in preparing the endometrium for implantation and sustaining early pregnancy is well established in ART practice [12]. The role of serum progesterone concentration measurement within ART cycles in determining the outcome of embryo transfer and as a guide for clinical decision-making is yet to be defined. Secondary analysis of four randomised trials showed inconsistent findings [7, 13]. Vuong et al. found that progesterone concentration greater than 1.14 ng/mL was associated with higher live births in the freeze-all group as compared to the fresh transfer group, which reflects the findings of previous observational studies [14]. The other secondary analysis of three large trials demonstrated frozen transfer associated with higher rates of live birth irrespective of progesterone concentration on the day of HCG trigger, with greater differences in live birth rates in patients with progesterone ≥ 1.14 ng/mL as compared to those with progesterone < 1.14 [13].

While these secondary studies explored the potential role of progesterone elevation in IVF cycle outcome, they have been underpowered for interaction analysis and they oftern excluded patients with advanced age due the inclusion criteria of the primary trials. Therefore, it would be important at this stage to use large observational data to explore such a research question, without restricting to young patients with good prognosis. This study aims to determine the potential role of progesterone as a biomarker, routinely examined in IVF treatment cycles, in guiding the selection of fresh or freeze-all embryo transfer strategy in unselected population as well as in patients over 35 years old.

## Methods

This was a single-centre retrospective cohort study conducted in accordance with the Declaration of Helsinki. Ethics approval for the study was granted by Chengdu Xinan Hospital Reproductive Medicine Ethics Committee (2021SZLS001) and Monash University Human Research Ethics Committee (23120). Informed consent was waived as it was a retrospective study based on routinely. The medical records of all patients undergoing IVF/ICSI treatment at Chengdu Xinan Hospital between January 2015 and September 2019 were reviewed. We included all patients who commenced their first fresh stimulated IVF/ICSI cycle using autologous gametes during this period. We excluded oocyte preservation and oocyte donation cycles, artificial insemination cycles, preimplantation genetic testing (PGT) cycles and donor sperm cycles. Cycles where oocyte retrieval was cancelled or no embryos were available for transfer were also excluded from the final analysis. To avoid confounding by indication, we also excluded those cycles where a freeze-all strategy was performed with absolute indication in our setting, with the serum progesterone on the trigger day ≥ 2.0 or ≥ 20 oocytes collected.

### Procedures and interventions

Protocols for treatment included both gonadotrophin releasing hormone (GnRH) agonist and antagonist protocols. All patients were monitored with ultrasound and serial hormone studies. Serum progesterone concentration was routinely measured during controlled ovarian hyperstimulation including measurement on the day of trigger. When 3 follicles were ≥ 18 mm, final oocyte maturation was triggered with the administration of human chorionic gonadotrophin (HCG) or GnRH agonist trigger and vaginal oocyte collection was performed 36 h later under sedation with transvaginal ultrasound guidance. Fertilisation was by conventional IVF or intracytoplasmic sperm injection (ICSI) depending on semen analysis results. Embryos were cultured in vitro to cleavage or blastocyst stage.

Luteal-phase support was commenced on the night of oocyte collection and continued up to the time of a positive pregnancy test. In patients undergoing frozen embryo transfer, transfer was performed in a natural cycle in patients who were normal ovulatory or otherwise in a hormone replacement (HRT) cycle.

### Outcome measure and definitions

The primary outcome of interest was live birth after 28-week gestation following the first embryo transfer and expressed as an odds ratio (OR).

### Statistical analysis

We used logistic regression to calculate the odds ratio (OR) with 95% confidence intervals (CI) of live birth in a freeze-all transfer strategy versus a fresh embryo transfer strategy. We selected a minimal set of confounding factors to be adjusted in the analysis, based on clinical knowledge, including female age, BMI, the diagnosis of PCOS and the number of oocytes and serum progesterone levels on the day of hCG triggering. We did not adjust for any potential mediators, including the number or stage of embryos transferred, as is the recommended methodology for statistical analysis in observational studies [15].

To evaluate whether the treatment effect varied with serum progesterone concentration, we built a multivariable fractional polynomial interaction test within a logistic regression model. This approach allows the detection of non-linear associations, in addition to common linear associations, and the selection of the best-fitting one based on the smallest Akaike information criterion. This methodology has been successfully applied in other biomarker interaction analyses in reproductive medicine [16]. We restricted the analyses to linear and first-degree fractional polynomials for both main effects and interaction due to clinical plausibility. The *p*-value for the interaction was based on a likelihood ratio test. We visualised (1) the treatment effect at different serum progesterone concentrations and (2) both the predicted live birth rates in a fresh and a freeze-all embryo transfer strategies at different serum progesterone concentrations.

To evaluate the robustness of the findings, we also divided the cohort into quartiles based on serum progesterone concentration and performed logistic regression within each quartile adjusting for the same covariates. We plotted the ORs and 95% CIs for each group in a forest plot. The main analysis was performed based on complete cases. We also performed multiple imputations in a sensitivity analysis to examine the robustness of the findings.

In addition, we performed sensitivity analyses on the multivariable fractional polynomial

interaction analysis for a subset of patients over 35 years old, a subset of patients

with a single embryo transfer and a third subset of patients with blastocyst transfer. All statistical analyses was performed in Stata v16.1 (StataCorp, College Station, TX, USA).

## Results

Over the study period, 43,576 patients commenced their first cycle of treatment. Patients with donor sperm (*n* = 1340), preimplantation genetic testing (*n* = 460), artificial insemination (*n* = 1) cycles, for oocyte donation (*n* = 60), oocyte cryopreservation (*n* = 29), no embryo available for transfer (*n* = 3297) or oocyte retrieval canceled (*n* = 1341) were excluded from the cohort. We then excluded patients not returning for frozen embryo transfer within the data collection period, with oocyte number ≥ 20, progesterone on the day of HCG was ≥ 2 nmol/mL or with triple embryo transfer. This left 26,661 patients included, of which 15,539 underwent fresh embryo transfer strategy and 11 122 underwent freeze-all embryo transfer strategy (Appendix 1).

The baseline characteristics of unselected patients undergoing fresh or freeze-all embryo transfer in their first cycle of treatment as displayed in Table 1. There were differences between the two groups with respect to age, number of oocytes retrived, frequency of PCOS diagnosis and serum progesterone concentration with these values all being higher in the freeze-all group. The proportion of couples undergoing treatment for primary infertility was higher in the fresh transfer group.

**Table 1:** Baseline characteristics

Characteristics	Freeze all (*N* = 11,122)	Fresh (*N* = 15,539)	*p*-value
Female age (years)	32.0 (5.1)	30.9 (4.1)	< 0.001
Body mass index (BMI) kg/m^2^	22.0 (3.0)	21.9 (3.0)	0.15
Primary infertility (%)	4770 (42.9%)	7079 (45.6%)	< 0.001
Duration of infertility	3 (2–6)	3 (2–5)	0.008
Polycystic ovary syndrome (PCOS)	988 (8.9%)	768 (4.9%)	< 0.001
Number of oocytes collected	10 (5–15)	9 (6–12)	< 0.001
Serum progesterone concentration on the trigger day (ng/mL)	1.06 (0.75–1.43)	0.98 (0.70–1.29)	< 0.001
Serum oestradiol concentration on the trigger day (pg/mL)	3235 (1762, 4854)	2692 (1756, 3851)	< 0.001
Serum luteinizing hormone concentration on the trigger day (IU/L)	1.66 (1.09, 2.62)	1.52 (1.02, 2.25)	< 0.001

All values are presented as mean and standard deviation where data are normally distributed or median and interquartile range where data are skewed

Compared to the fresh transfer group, the freeze-all group had more single embryo transfers (21.8% vs 7.5%), more blastocyst transfers (63.1% vs 13.9%), higher clinical pregnancy rate (56.4% vs 50.3%) and higher live birth rate (43.9% vs 40.3%) (Table 2).

**Table 2:** Outcomes following freeze-all or fresh embryo transfer

Outcomes	Freeze all (*N* = 11,122)	Fresh (*N* = 15,539)	*p*-value
Number of embryos			< 0.001
Single embryo transfer	2420 (21.8%)	1168 (7.5%)	
Double embryos transfer	8688 (78.2%)	14373 (92.5%)	
Stage of embryos			< 0.001
Clevage	4101 (36.9%)	13382(86.1%)	
Blastocyst	7008 (63.1%)	2157 (13.9%)	
Clinical pregnancy	5270 (56.4%)	7817 (50.3%)	< 0.001
Live birth	4883 (43.9%)	6256 (40.3%)	< 0.001
Multiple birth	1560 (14.0%)	1298 (8.4%)	< 0.001

**Fig. 1:**
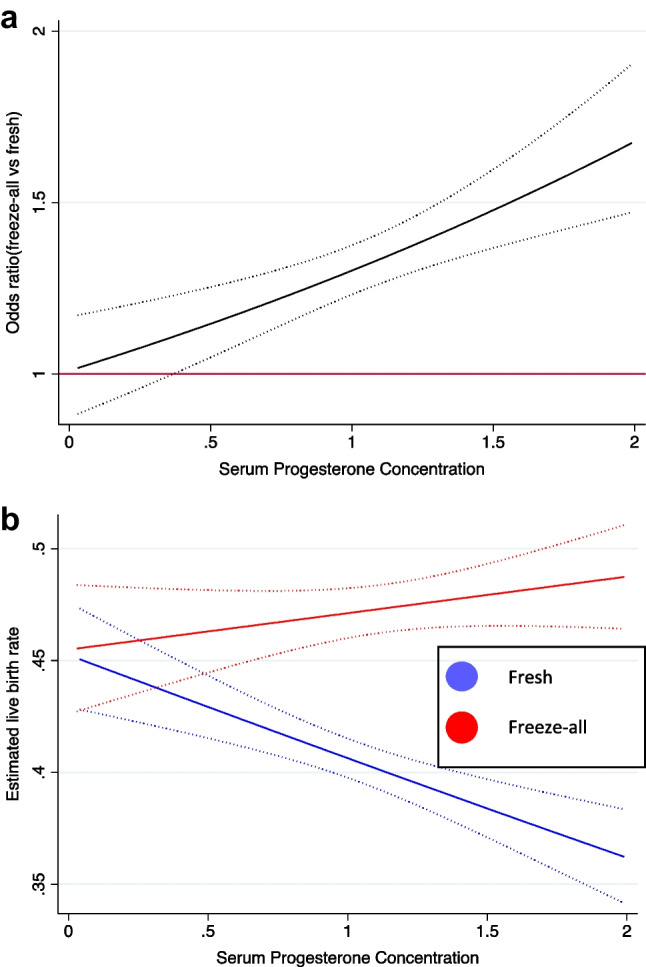
**a** Odds ratio of live birth for freeze-all vs frozen transfer at different serum progesterone concentrations. **b** Predicted live birth for freeze-all and fresh embryo transfer by progesterone concentration on HCG trigger day

In multivariable fractional polynomial interaction analysis, a linear polynomial (i.e. power=1) was chosed for progesterone concentration as it had the smallest Akaike information criterion. After adjusting confounding factors considering non-linearity (female age (power = 3), BMI (power = 1), the diagnosis of PCOS (power = 1) and the number of oocytes (power = − 0.5)), the multivariable fractional polynomial interaction analysis showed a positive interaction between serum progesterone concentrations and the choice of a freeze-all strategy versus fresh embryo transfer on live birth (*p* for interaction 0.0001). The predicted odds of live birth were greater in the frozen embryo transfer group at all progesterone concentrations. The difference in live birth rate between groups was demonstrated at all progesterone concentrations but the effect was more pronounced with a progressive increase in progesterone concentration (Fig. 1a, b). This relationship was maintained in both unadjusted and adjusted models. When performing multiple imputation for covariates with missing data, the interaction effect was still valid (*p* for interaction < 0.0001).

**Fig. 2:**
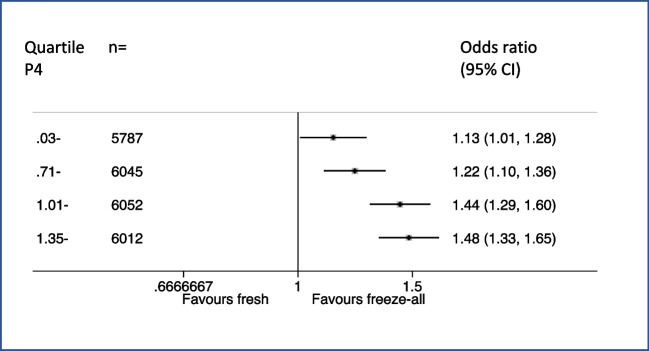
Forest plot for adjusted odds ratio based on progesterone concentration (quartile)

When further stratify progesterone concentrations into four quartiles, the calculated odds ratios of freeze-all versus fresh transfer on live birth were higher groups with higher progestereone concentrations (Fig. 2), consistent with findings from multivariable fractional polynomial interaction analysis in both unadjusted and adjusted models.

**Fig. 3:**
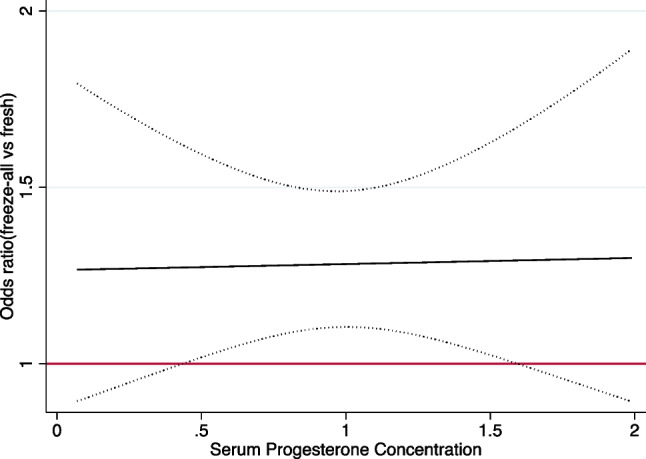
Odds ratios of live birth for freeze-all vs fresh transfer at different serum progesterone concentrations in patients over 35 years old

When repeating the multivariable fractional polynomial interaction analyses in a subset of patients with single embryo transfer (*n* = 3586) or blastocyst transfer (*n* = 9107), the interaction between progesterone and treatment effect was still present (Supplementary Figure 1 and 2, *p* for interaction = 0.0423 and 0.0025 respectively). For patients over 35 years old (*n* = 4687), the positive interaction between progesterone and treatment effect was also present although the treatment effect curve is almost flat (Fig. 3, *p* for interaction = 0.01).

## Discussion

Our results suggest that in an unselected population undergoing IVF/ICSI treatment, there is an increased chance of pregnancy associated with a freeze-all embryo transfer strategy at different progesterone concentrations on HCG trigger day. The effect appears to be dose related with an increasing magnitude of effect with rising progesterone concentration. Such an interaction was also observed in subsets of patients with single embryo transfer, blastocyst ransfer and patients over 35 years old, but the benefit of a freeze-all policy appears small in patients over 35 years old.

The strength of the study includes a large sample size as well as the exclusion of patients with absolute indications of freeze-all transfers to minimise indication bias. Nevertheless, there are a few limitations. Our study was retrospective in design and limited to a single centre. Although the single centre has a large and geographically varied population, the results may not be readily generalisable to all centres and patient populations. While we did adjust for a minimal set of confounders in the final analysis, one of the inherent limitations of our study design is the inability to adjust for unknown confounders. At our centre, vitrification was the preferred method of embryo cryopreservation and our findings may not, therefore, be comparable to other programs where methodology other than vitrification-thaw is used in freeze-all cycles. The success of freeze-all transfer is defined by an efficient and well-established freezing program. The pregnancy rate amongst our freeze-all cycles was 43 percent which is comparable to large registry data [17, 18].

These limitations notwithstanding, the results we have presented would suggest that in an unselected patient population, a freeze-all approach may be associated with improved pregnancy outcome as well as significantly reducing the risk of OHSS and adverse perinatal outcome related to controlled ovarian hyperstimulation effect on the endometrium and placentation [19, 20]. We did not include cycles in which the progesterone on the day of trigger was > 2 ng/mL to avoid confounding by indication but modelling would suggest that this subgroup of patients would derive greater benefit from this approach. Our findings are consistent with the previous large secondary analyses examining the role of progesterone in fresh and frozen transfer [13]. In comparison to the primary studies included in Yu et al. which were restricted to relatively good prognosis populations, we included all couples undergoing treatment in their first cycle suggesting that a freeze-all approach would benefit a wider population group. By comparison, our study population was older, more likely to have primary infertility and had fewer oocytes collected. It may be that there is an intrinsic benefit in fresh over frozen transfer in poorer prognosis populations due to the impact of cryo-storage on those embryos and a secondary analysis in couples with female age greater than 35 years is planned to explore this further. The reduction of benefit in women aged greater than 35 years may also be representative of an interaction of embryo quality and endometrial receptivity to achieve a successful pregnancy and a relatively greater impact of elevated progesterone concentration in women aged less than 35 where we would assume superior embryo quality. It is also possible that the effects of supraphysiological sex steroid levels on the endometrium associated with optimal ovarian response have an increasingly detrimental effect on endometrial receptivity in fresh transfer cycles. We would aim that our study forms a basis for further investigation and that the hypothesis be tested in future individual participant data meta-analysis and confirmed in other populations and settings [21]. Such analyses may be used to explore progesterone and other biomarkers which may direct a personalised approach to embryo transfer and other components of assisted reproductive treatment cycles. More recently, there have been studies investigating the impact of serum hormone levels on the day of transfer on fertility outcomes, but the findings are conflicting [22, 23] and it is unclear whether these biomarkers on the day of transfer could better guide the clinical choice on the transfer strategy. Therefore, these should be further investigated in future studies.

## Conclusion

In couples undergoing their first IVF cycles, there is a benefit from a freeze-all transfer strategy as compared to fresh at all serum progesterone concentrations. The effect on live births may be of increasing magnitude with increasing progesterone concentration. In patients over 35 years old, the benefit of a freeze-all policy appears small. While this study is hypothesis-generating, it suggests that progesterone may represent a valuable biomarker to guide clinical decision-making and optimise outcomes for couples undergoing assisted reproductive treatment.

### Supplementary information

ESM 1 (DOCX 152 kb)

## Data Availability

Data are available from the authors upon reasonable request and with permission of the research office and ethics committee at Chengdu Xinan Gynecology Hospital.
