# Supplementary material for: Can serum progesterone concentration direct a fresh or freeze-all transfer strategy in the first in vitro fertilisation cycle?
Source: J Assist Reprod Genet. 2024 Apr 3;41(6):1549–55. doi: 10.1007/s10815-024-03103-y (PMC11224202; doi:10.1007/s10815-024-03103-y)
Supplement: Supplementary file 1 — (DOCX 152 kb) [file 10815_2024_3103_MOESM1_ESM.docx]

**Appendix 1 : Flowchart for patient selection**

43577 patients

undergoing their first IVF cycle

Excluded first cycles:

Oocyte cryopreservation n=29

Oocyte donation n=60

Artificial insemination n=1

PGT cycles n=460

Donor sperm n=1340

Cancelled oocyte retrieval n=1341

No transferrable embryos n=3297

Excluded cycles:

Not returing for frozen transfer n=3484

Oocyte number >20 n=4319

Progesterone ≥2.0 nmol/L n= 2561

Triple embryo transfer n=23

37049 patients

26661 patients

**Supplementary Figure 1 Odds ratios of live birth for freeze all vs frozen transfer at different serum progesterone concentrations in patients with single embryo transfer**

**Supplementary Figure 2 Odds ratios of live birth for freeze all vs frozen transfer at different serum progesterone concentrations in patients with blastocyst transfer**
